# A High Stability Time Difference Readout Technique of RTD-Fluxgate Sensors

**DOI:** 10.3390/s17102325

**Published:** 2017-10-12

**Authors:** Na Pang, Defu Cheng, Yanzhang Wang

**Affiliations:** 1 College of Instrumentation & Electrical Engineering, Jilin University, No. 938 Ximinzhu Street, Changchun 130026, China; pangna505@163.com (N.P.); yanzhang@jlu.edu.cn (Y.W.); 2 College of Information Technology and Media, Beihua University, No. 3999 East Binjiang Road, Jilin 132013, China

**Keywords:** RTD-fluxgate, negative magnetic saturation time, readout scheme, time difference

## Abstract

The performance of Residence Times Difference (RTD)-fluxgate sensors is closely related to the time difference readout technique. The noise of the induction signal affects the quality of the output signal of the following circuit and the time difference detection, so the stability of the sensor is limited. Based on the analysis of the uncertainty of the RTD-fluxgate using the Bidirectional Magnetic Saturation Time Difference (BMSTD) readout scheme, the relationship between the saturation state of the magnetic core and the target (DC) magnetic field is studied in this article. It is proposed that combining the excitation and induction signals can provide the Negative Magnetic Saturation Time (NMST), which is a detection quantity used to measure the target magnetic field. Also, a mathematical model of output response between NMST and the target magnetic field is established, which analyzes the output NMST and sensitivity of the RTD-fluxgate sensor under different excitation conditions and is compared to the BMSTD readout scheme. The experiment results indicate that this technique can effectively reduce the noise influence. The fluctuation of time difference is less than ±0.1 μs in a target magnetic field range of ±5 × 10^4^ nT. The accuracy and stability of the sensor are improved, so the RTD-fluxgate using the readout technique of high stability time difference is suitable for detecting weak magnetic fields.

## 1. Introduction

The fluxgate sensor has been widely used in geomagnetic observation, space magnetic field measurement, and other fields due to its high sensitivity, small size, and low power consumption, etc. [1,2,3,4,5]. The RTD-fluxgate sensor developed by Bruno Andò, et al., using the hysteresis saturation phenomenon of soft magnetic material, can detect magnetic fields through the corresponding relationship between the residence times difference of the induction pulse signal and the target magnetic field [6,7,8], and has the advantages of a simple detection procedure, strong anti-interference ability, easy miniaturization, and digitization, etc. It has attracted more attention in the fields of national defense military and geomagnetic prospecting [9,10,11]. However, the noise of the induction signal makes the time difference read uncertain, seriously affecting the accuracy of RTD-fluxgate sensor measurements [12,13].

The quality of the induction signal is closely related to variations in the dynamic permeability of the magnetic core [14,15]. To reduce the effects of noise, an effective approach is utilized with a 2714A annealed core with a sharp hysteresis loop and low coercive field [16,17]. Because the induction pulse signal corresponds to the state of magnetic saturation, Bruno Andò, et al. read the time difference between the peak points of the induction pulse signal to measure the target magnetic field [18,19]. As a result of the sensitivity of the RTD-fluxgate unit to repetitive magnetization, magnetic core noise, electronic circuit noise, and environment interference, it is difficult to locate the peak points accurately [20,21,22]. Wang Y.Z., et al. read the time difference by using the threshold which is set slightly lower than the peak value of the induction signal [23]. Although the error of locating the peak points can be avoided, the threshold has to be set. Even in this case, the magnetic noise and electrical noise cause the transverse instability of the induction signal, resulting in the uncertainty of the time difference readout. Lu S.B., et al. fitted the pulse curve by using the data near the peak value of positive and negative output pulse, and depending on the time of three adjacent peaks to calculate the residence time difference which measure the target magnetic field [24]. The method does not need to consider the influence of the threshold set on the output performance of the sensors, however, the accuracy of curve fitting is limited due to noise interference existing in the induction signal. In order to reduce the influence of noise on time difference reading, several approaches for filtering the induction signal are introduced [25,26]. Although the method can reduce the noise intensity, it is mainly aimed at detecting the amplitude of the signal. After filtering, there is a certain degree of distortion in the induction signal which causes the time difference reading error. Therefore, the induction signal filtering is limited to time difference reading. According to RTD-fluxgate detection theory, the large deviation in the output time difference can be caused by output signal noise. If the induction signal is used individually to read time difference, the noise effects cannot be avoided.

In order to improve the accuracy and stability of RTD-fluxgate sensors and reduce the noise that produces uncertainties in the estimation of the residence times, the relationship between the state of the magnetic core and the target magnetic field is studied. On the basis of analyzing the working principle of the RTD-fluxgate sensor, in this paper, a new method of time difference reading between the excitation signal passing through the zero point as a reference time and the negative output pulse is proposed, that is, the excitation signal and the output pulse signal are combined to read the negative magnetic saturation time Δ*T_NMST_* as the detection quantity to measure the target magnetic field. A mathematical model of the sensor output response between the Δ*T_NMST_* and (DC) target magnetic field *H_x_* is established under a triangular excitation signal. It is analyzed that Δ*T_NMST_* and sensitivity *S_NMST_* change with the variation of the amplitude and frequency of the excitation current. The theoretical and experimental comparison between the NMST and the BMSTD readout strategy is presented and discussed. The results show that this method can reduce the influence of the output pulse noise effects on the readout technique.

The rest of this paper is organized as follows. Section 2 presents the working principle of RTD-fluxgate sensor in the case of triangular signal excitation. The influence of the output induction signal noise on the time difference readout strategy is analyzed. In Section 3, the NMST readout strategy is introduced and the uncertainty of the method is calculated. By using the NMST readout strategy, the mathematical model of the sensor output response is established and the variation of Δ*T_NMST_* and *S_NMST_* with different excitation conditions is analyzed. In Section 4, experiments are investigated to check the performance of the NMST readout strategy compared to the BMSTD readout strategy. Section 5 concludes the whole paper and the results are presented.

## 2. Working Principle of RTD-Fluxgate Sensors and Stability Analysis of Time Difference Detection

### 2.1. Working Principle of RTD-Fluxgate Sensors under Triangular Excitation Signal

**Figure 1:**
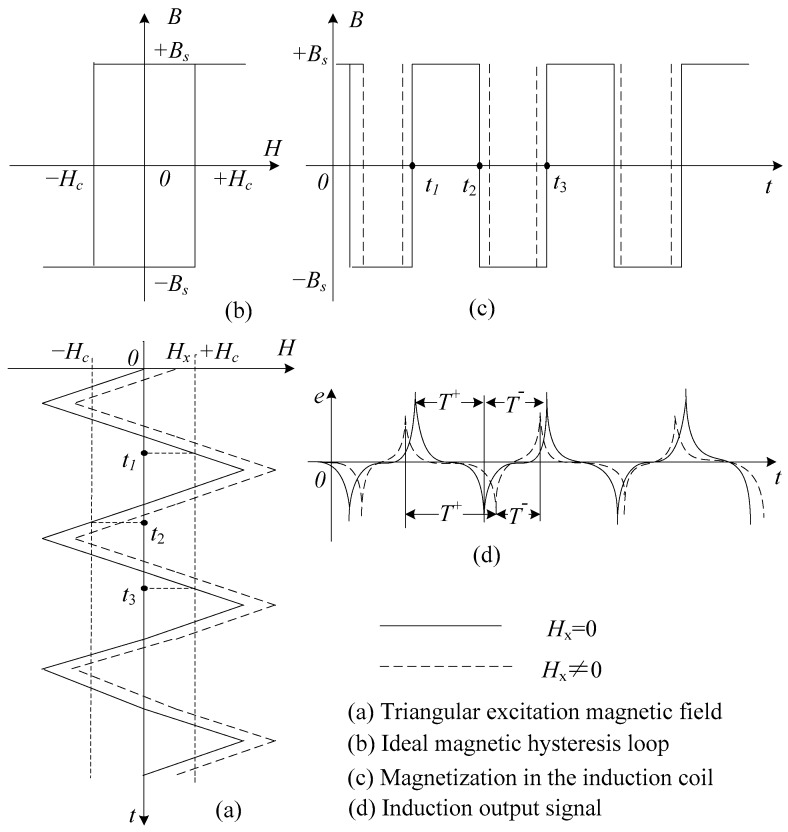
Schematic diagram of residence times difference (RTD)-fluxgate working principle.

The magnetic core of the sensor is magnetized by a periodically alternating triangular magnetic field to the states of two-way over-saturation, as is shown in Figure 1a. The ideal hysteresis loop of the magnetic core is shown in Figure 1b. The magnetization produced in the induction coil is shown in Figure 1c. If a target magnetic field *H_x_* exists along the axis of the sensor, the residence times of the magnetic core in positive and negative saturation states are different. Because the time interval between the positive and negative pulse of induction signal *T*^+^ is not equal to the time interval between the negative pulse and the next positive pulse *T*^−^, a time difference between them exists. We may obtain the values of *H_x_* by detecting the bidirectional magnetic saturation time difference △*T* = *T*^+^ − *T^−^* of the output pulse signal which relates to the states [8,27,28,29], as is shown in Figure 1d.

In this article, the case of triangular excitation is considered. The triangular excitation is assumed to have amplitude and period equal to *H_m_* and *T_e_*, respectively. The expression is as follows:

(1) $$H_{e}(t)=\{\begin{array}{ll} \alpha t & NT_{e}-\frac{T_{e}}{4}<t<NT_{e}+\frac{T_{e}}{4}\\ -\alpha (t-\frac{T_{e}}{2}) & NT_{e}+\frac{T_{e}}{4}<t<NT_{e}+\frac{3T_{e}}{4} \end{array}$$

As is shown in Figure 1a, when the excitation field *H_e_*(*t*) reaches saturation of magnetic core, the times are *t*_1_, *t*_2_ and *t*_3_. At period *T_e_* of the induction signal, it is straightforward to calculate the residence times *T*^+^ and *T*^−^:

(2) $$T^{+}=t_{2}-t_{1}=\frac{2H_{x}}{\alpha }+\frac{T_{e}}{2}$$

(3) $$T^{-}=t_{3}-t_{2}=-\frac{2H_{x}}{\alpha }+\frac{T_{e}}{2}$$

The output response of the RTD-fluxgate under the triangular excitation field is expressed as shown in Equation (4):

(4) $$\Delta T=T^{+}-T^{-}=\frac{4H_{x}}{\alpha }=\frac{4H_{x}}{4H_{m}f_{e}}=\frac{H_{x}}{H_{m}f_{e}}$$

The sensitivity of the RTD-fluxgate can be estimated:

(5) $$S=\frac{\partial \Delta T}{\partial H_{x}}=\frac{4}{\alpha }=\frac{1}{H_{m}f_{e}}$$

### 2.2. Stability Analysis of BMSTD Readout Technology

**Figure 2:**
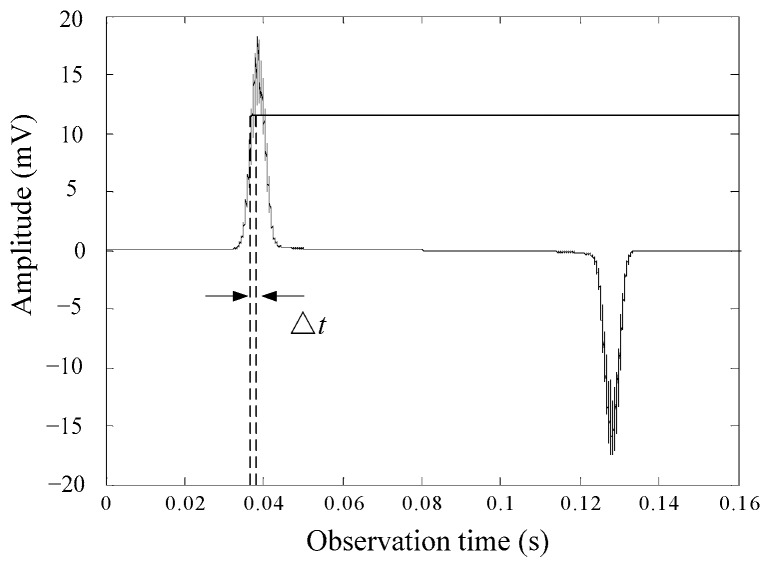
Noise affects the induction output signal.

Adoping the detection method using the induction output signal’s hysteresis shape and timing, that is, counting the low and high levels formed after the signal is amplified and then made in to shapes, the RTD-fluxgate can read Δ*T*. According to the method, when the excitation condition and the core material are determined, the stability of the time difference measurement is only related to the readout technology. Generally, the output signal is not smooth, and there is transverse instability because of electrical noise, magnetic noise, etc. As is shown in Figure 2, the trigger position of the output signal varies because of the noise, eventually leading to fluctuation of the time difference.

**Figure 3:**
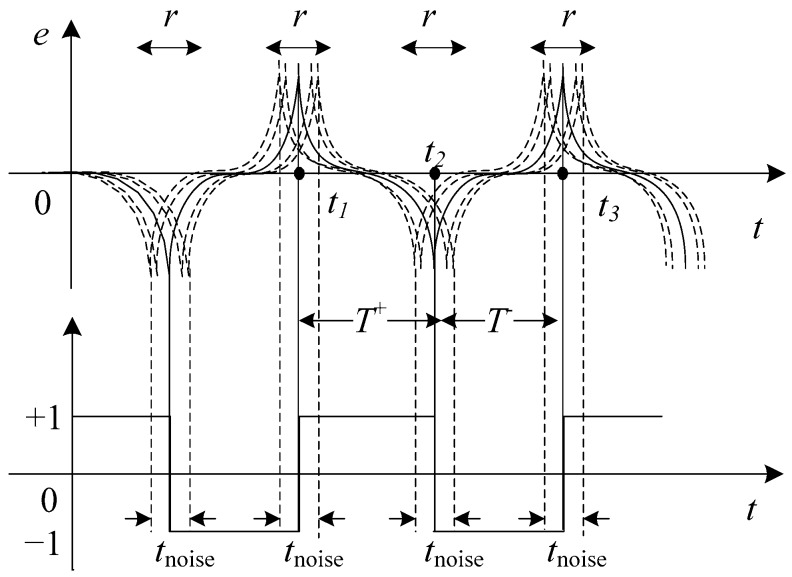
Noise effects in the bidirectional magnetic saturation time difference (BMSTD) readout.

It is assumed that in an ideal case, the induction output signal does not have noise interference. The time of the first positive pulse appears as *t*_1_, the time of the first negative pulse appears as *t*_2_, and the second positive pulse appears as *t*_3_. The presence of magnetic and electrical noise affect the estimation of three transition times, so the corresponding actual transition times are $t_{1}^{\prime }$, $t_{2}^{\prime }$, $t_{3}^{\prime }$, respectively. As is observed in Figure 3, the solid line represents the ideal output residence times, and the dotted line represents the actual output residence times when influenced by noise. The *t*_noise_ is an uncertainty product due to noise in the estimation of the residence times. Δ*T* is expressed as follows:

(6) $$\Delta T=T^{+}-T^{-}=(t_{2}^{\prime }-t_{1}^{\prime })-(t_{3}^{\prime }-t_{2}^{\prime })=2t_{2}^{\prime }-t_{1}^{\prime }-t_{3}^{\prime }$$

The magnetic noise is not related to the electrical noise in the detection system, therefore, the uncertainty of Δ*T* is affected by noise, which is described in Equation (7):

(7) $$\gamma \approx \sqrt{4(\gamma_{m2}^{2}+\gamma_{e2}^{2})+(\gamma_{m1}^{2}+\gamma_{e1}^{2})+(\gamma_{m3}^{2}+\gamma_{e3}^{2})}$$

In Equation (7), *γ* represents the total noise of Δ*T*, *γ_mi_* represents the magnetic noise of transition time *t_i_*, and *γ_ei_* represents the electrical noise of transition time *t_i_*. Assuming the same uncertainty value for each *t_i_*, it is possible to write the following expression:

(8) $$\gamma \approx \sqrt{6(\gamma_{mi}^{2}+\gamma_{ei}^{2})}$$

By using the BMSTD readout scheme, three transition times need to be estimated. Because each transition time is affected by noise, the uncertainties of Δ*T* are fairly large. In view of the situation above, in order to minimize the influence of noise on the detection and reduce the uncertainty of the time difference, the readout technology needs to be improved.

## 3. The Mathematical Output Response Model of the NMST Readout Technique and Stability Analysis

### 3.1. The NMST Readout Technique and the Mathematical Output Response Model

If a target magnetic field *H_x_* exists, the time when the soft magnetic material reaches the positive and negative saturation states will change. The time when the soft magnetic material reaches two steady points of the double-well potential are different. The *H_x_* directly affects the time when the magnetic core reaches the positive and negative saturation states. Therefore, the time of magnetic core saturation is equivalent to the bidirectional magnetic saturation time difference, Δ*T*, which can be considered as the detection quantity to measure *H_x_*.

**Figure 4:**
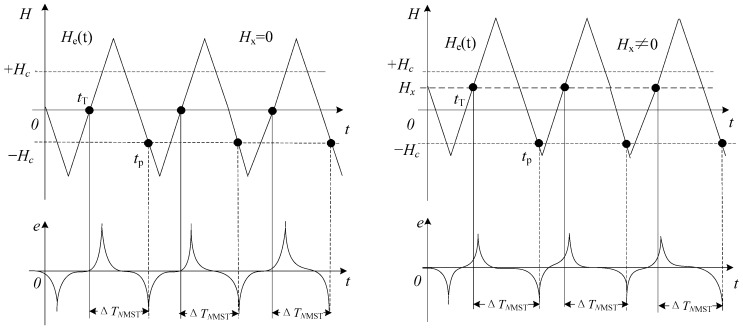
Diagram of the negative magnetic saturation time NMST readout strategy.

In this paper, however, we also present a different way to process the excitation and induction signals to get information on the target field. This readout strategy is quite similar to the BMSTD scheme except for the use of a reference time. It is known when the excitation signal passes through the zero point, and so the transition time is used as reference time. The method of reading the time difference between the reference time and the negative output pulse is proposed, that is, the excitation signal and induction signal are combined to read the negative magnetic saturation time, Δ*T_NMST_*, to detect the *H_x_*. As is shown in Figure 4, when the magnetic core becomes saturated, an output pulse signal is generated on the induction coil; the transition time when the excitation signal amplitude is zero is used as the reference time, *t_T_*, and when the applied magnetic field exceeds the coercive field, −*H_c_*, the induced voltage produces a negative pulse at *t_P_*. The relationship between the triangular excitation field *H_e_*(*t*) expressed by Equation (1) and the target field *H_x_* is as follows:

(9) $$\begin{array}{cc} t_{T}: & \alpha t_{T}=0 \end{array}$$

(10) $$\begin{array}{cc} t_{P}: & H_{x}-\alpha (t_{p}-\frac{T_{e}}{2})=-H_{c} \end{array}$$

Deduced by Equations (9) and (10):

(11) $$t_{T}=0$$

(12) $$t_{p}=\left(H_{c}+H_{x}\right)/\alpha +\frac{T_{e}}{2}$$

The time difference Δ*T_NMST_* between *t_P_* and *t_T_* is defined to negative magnetic saturation time, which is given by:

(13) $$\Delta T_{NMST}=t_{p}-t_{T}$$

When using the NMST readout strategy, the output response of the RTD-fluxgate is as follows:

(14) $$\Delta T_{NMST}=t_{p}-t_{T}=\frac{\left(H_{c}+H_{x}\right)}{4H_{m}f_{e}}+\frac{T_{e}}{2}$$

The sensitivity expression for the NMST strategy obtained by using similar calculations is shown in Equation (15):

(15) $$S_{NMST}=\frac{\partial \Delta T_{NMST}}{\partial H_{x}}=\frac{1}{\alpha }=\frac{T_{e}}{4H_{m}}=\frac{1}{4H_{m}f_{e}}$$

**Figure 5:**
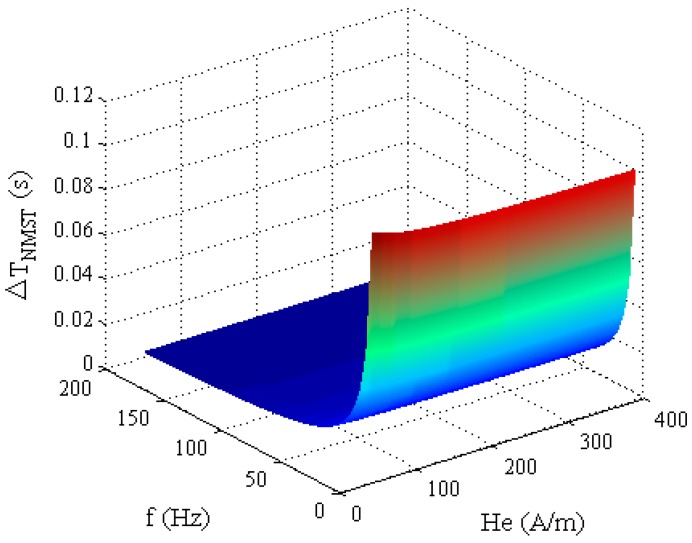
The relationship between Δ*T_NMST_* and *H_e_*(*t*).

When *H_x_* and the coercive field *H_c_* of the magnetic core are fixed, the relationship between Δ*T_NMST_* and *H_e_*(*t*) is as shown in Figure 5. When the amplitude *H_m_* and the frequency *f* of the excitation magnetic field are smaller, the Δ*T_NMST_* is greater.

**Figure 6:**
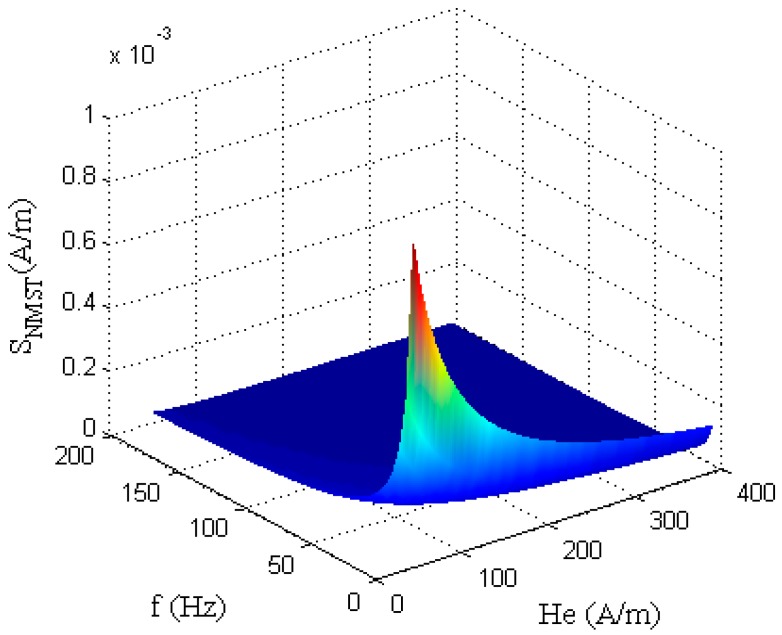
The relationship between *S_NMST_* and *H_e_*(*t*).

According to Equation (15), the variation tendency of the sensitivity of the RTD-fluxgate *S_NMST_*, obtained by using the NMST readout strategy, is shown in Figure 6.

From the figure above, when using the NMST readout technology, the *S_NMST_* of RTD-fluxgate is inversely proportional to the excitation magnetic field’s amplitude *H_m_* and frequency *f*. Therefore, when the excitation circuit structure does not need changing, the sensitivity of the RTD-fluxgate is improved by reducing the excitation magnetic field amplitude *H_m_* and the frequency *f*, and at the same time, the power consumption is cut down. But, according to the working principle of RTD-fluxgate, the magnetic core of the sensitive unit needs to achieve bidirectional oversaturation, so the excitation magnitude of *H_m_* should at least saturate the core and the excitation frequency *f* is too low, which will lead to a smaller range of the measured magnetic field and a worse effect of the induction output signal. Therefore, the excitation parameters can be determined according to the actual measurement conditions.

### 3.2. Stability Analysis of NMST Readout Technology

**Figure 7:**
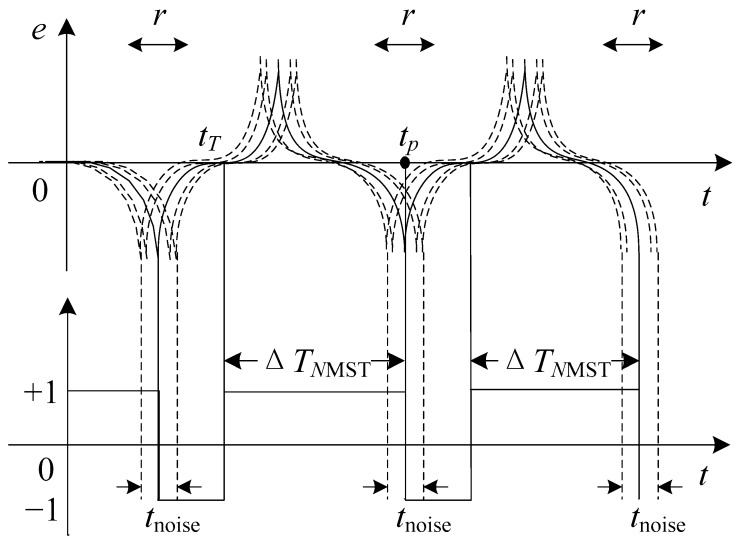
Noise effects in the NMST readout scheme.

When using the NMST readout scheme, the excitation signal is generated by the signal generator. The reference time *t_T_* does not need measuring, therefore *t_T_* can be obtained accurately. As is observed in Figure 7, in the ideal condition, because the output signal does not have noise interference, the time of the first negative pulse appears at *t_p_* and the corresponding actual transition time is *t_p_*’. The solid line represents the ideal output residence times and the dotted line represents the actual output residence times influenced by noise. An expression about Δ*T_NMST_* actually measured is as follows:

(16) $$\Delta T_{NMST}={t_{p}}^{\prime }-t_{T}$$

Because of the known *t_T_*, the noise affects the Δ*T_NMST_* at the transition time *t_p_*′. In this case, the presence of noise affects only the estimation of one transition time (the reference time being assumed to be noiseless) instead of three such times in the BMSTD strategy. The uncertainty of Δ*T_NMST_* affected by the noise is as follows:

(17) $$\gamma_{NMST}\approx \sqrt{\gamma_{mi}^{2}+\gamma_{ei}^{2}}$$

Based on analysis of theory, the relationship between the NMST and BMSTD readout schemes affected by noise is shown in the Equation (18). The NMST readout scheme can reduce the influence of noise on reading the time difference. Therefore, reading Δ*T_NMST_* to measure *H_x_* can improve the stability of the time difference.

(18) $$\gamma_{NMST}=\frac{1}{\sqrt{6}}\gamma$$

## 4. Experiments and Preliminary Results

**Figure 8:**
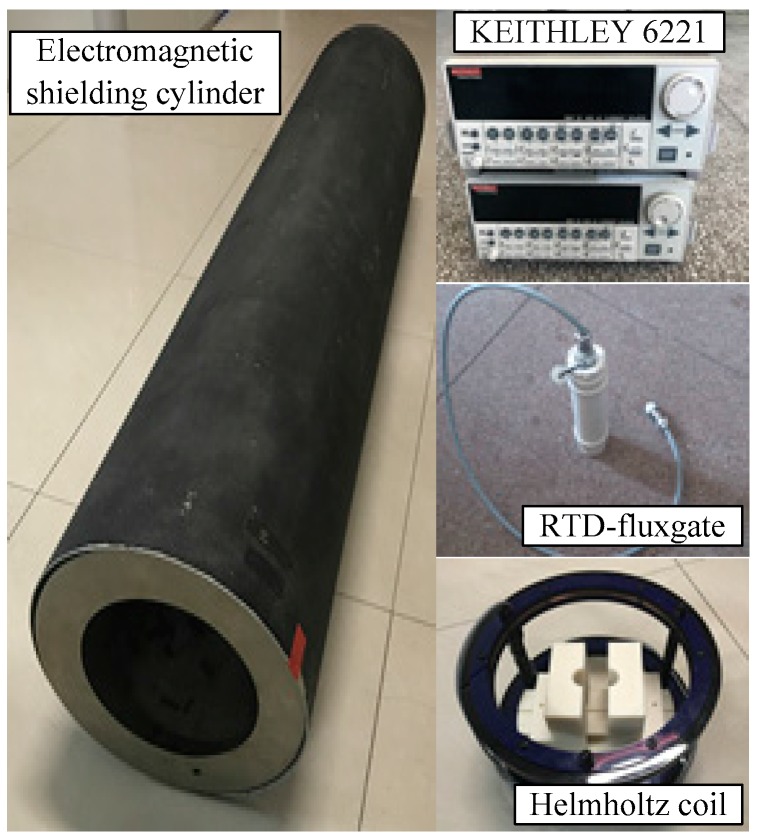
Experimental instruments.

**Figure 9:**
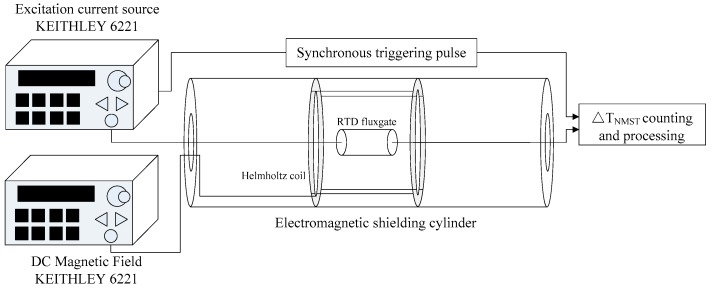
Experimental measurement schematic diagram.

The experimental instruments are shown in Figure 8. The RTD-fluxgate sensor is made by the Key laboratory of geophysical exploration equipment, Ministry of Education (Jilin University) and included two parts: the sensitive unit and the signal detection circuit. The sensitive unit consists of an excitation coil, magnetic core, and induction coil. The core adopts a co-based amorphous ribbon which is 0.8 mm in width, 0.025 mm in thickness, and 100 mm in length. The core is placed inside the non-magnetic framework. The excitation coil is symmetrically twined on both ends of the non-magnetic framework with the same number of turns, and the induction coil is twined in the middle. The excitation coil and induction coil are 100 turns and 1000 turns, respectively, using 0.1 mm enameled copper wires. The induction signal is amplified and rectified in the signal detection circuit, and then the rectangular signal is input to the time difference counting and processing part which is made up of Field Programmable Gate Array (FPGA) and STM32 microcontroller. In the magnetic shielding room made of multilayer silicon steel, the Helmholtz coil is placed in the middle of the multilayer electromagnetic shielding cylinder made of permalloy. The RTD-fluxgate is laid in the center of the loop, which can be considered as a homogeneous magnetic field. Two precision current sources of KEITHLEY 6221 are utilized in the experiment to excite the Helmholtz coil for generating a DC target magnetic field and drive the excitation coil of the RTD-fluxgate. The experimental measurement schematic diagram is shown in Figure 9.

The excitation coil of the sensitive unit generates a triangular excitation magnetic field. The induced voltage generated by the induction coil passes through the instrumentation amplifier circuit, the second level amplifier circuit, the addition circuit, and the shaping circuit, obtaining a rectangular signal which carries the information of the *H_x_*. The signal is input to the CH1 channel of FPGA logic signal processor. Regulating the excitation current source generates a synchronous triggering pulse. When the excitation voltage amplitude is zero, the trigger point is set. The synchronous trigger pulse is input to the CH2 channel of FPGA. FPGA uses two channel signals to count the number of time points when the counting frequency *f*_c_ is 100 MHz. The time points *N* is transmitted into Δ*T_NMST_* which is sent to STM32 for storage.

**Figure 10:**
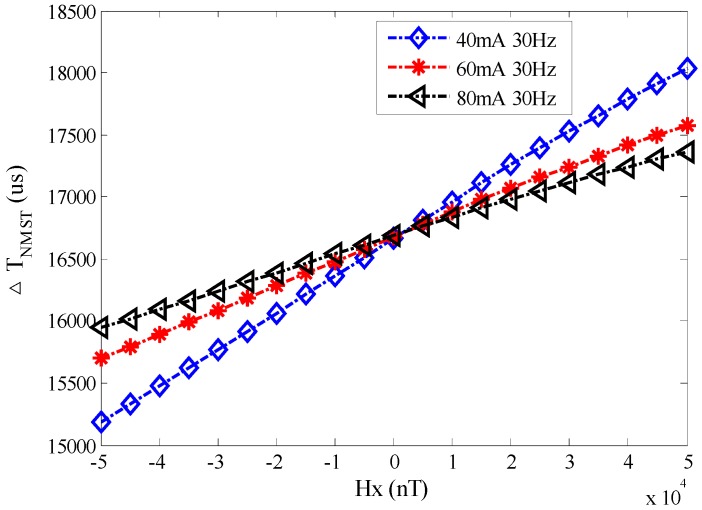
The curve between *H_x_* and Δ*T_NMST_* with different excitation currents.

(1) Relationship between the *H_x_* and Δ*T_NMST_*
  (a) The experiments are performed in the following conditions of different triangular excitation magnetic fields (excitation current from 40 mA to 80 mA with a 20 mA interval and excitation frequency at 30 Hz) and a range of *H*_x_ from −5 × 10^4^ nT to 5 × 10^4^ nT with a 5 × 10^3^ nT interval. Figure 10 shows the output time difference, Δ*T_NMST_*, of the RTD-fluxgate which are actually measured with different excitation currents.

From the figure above, the measured Δ*T_NMST_* and *H_x_* are linear. The linear regression technique (least square method) is used to fit the Δ*T_NMST_* curve. Assuming the linear fitting polynomial is *y* = *ax* + *b* (*a* ≠ 0) by using *n* data (*x*_i,_ *y*_i_) (*I* = 1, 2, … *n*), the sum of the deviation square between data points and the fitted curve is shown in below:

(19) $$d^{2}=\sum_{i=1}^{n}{\left[y_{i}-(ax_{i}+b)\right]}^{2}$$

**Figure 11:**
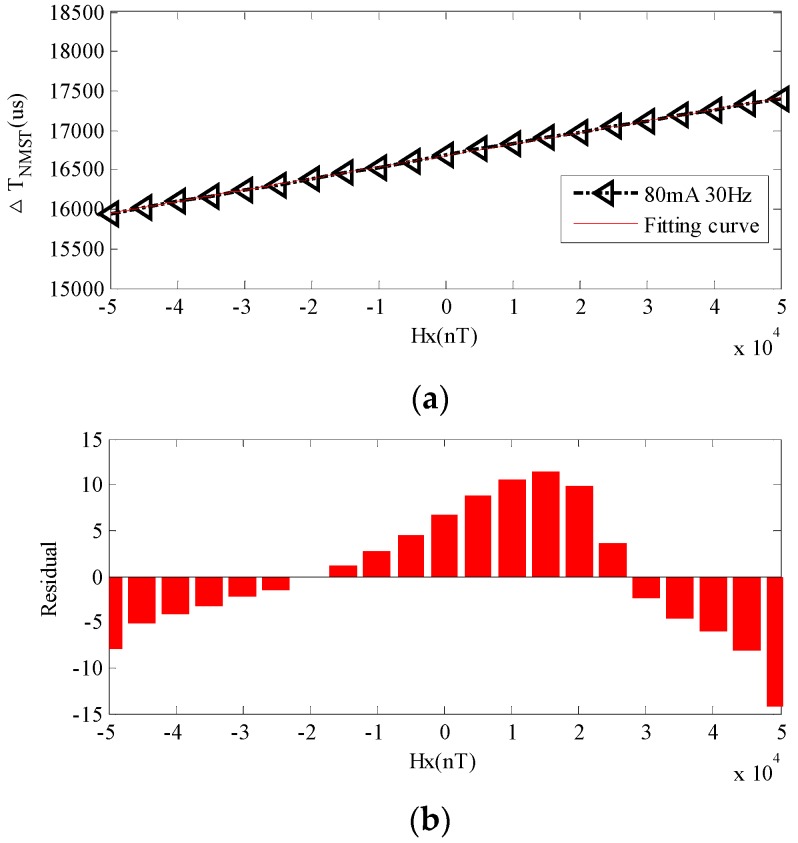
Fitting linearity deviations of Δ*T_NMST_*_1_ with *I*_1_ = 80 mA and *f* = 30 Hz. (**a**) Fitting curve of Δ*T_NMST_*_1_ ; (**b**) Deviations of Δ*T_NMST_*_1_.

One of the curves in Figure 10 is taken to illustrate the concept. When *d*^2^ = min(*d*^2^), the fitting curves between Δ*T_NMST_*_1_ and *H*_x_ with excitation current *I*_1_ = 80 mA and excitation frequency *f* = 30 Hz are presented Equation (20). The fitting linear deviations are shown in Figure 11, which shows that the linear deviations are mainly concentrated at both ends and center, so it is in accordance with the regulation of the linear sensor. The sum of the relative deviations square is 0.0573 and the RTD-fluxgate possesses good linearity in the whole range of measurement.

Δ*T_NMST_*_1_ = 0.0144 × *H_x_* + 1.67 × 10^4^ (20)

By using the same method, the fitting curves between Δ*T_NMST_* and *H_x_* with different excitation currents *I*_2_ = 60 mA and *I*_3_ = 40 mA are as follows:

Δ*T_NMST_*_2_ = 0.0191 × *H_x_* + 1.67 × 10^4^ (21)

Δ*T_NMST_*_3_ = 0.0289 × *H_x_* + 1.67 × 10^4^ (22)

From the Equations above, the sensitivities of different excitation currents are *S_NMST_*_1_ = 0.0144 μs/nT, *S_NMST_*_2_ = 0.0191 μs/nT and *S_NMST_*_3_ = 0.0289 μs/nT. When the excitation amplitude *H_m_* is smaller, the sensitivity *S_NMST_* is greater.

**Figure 12:**
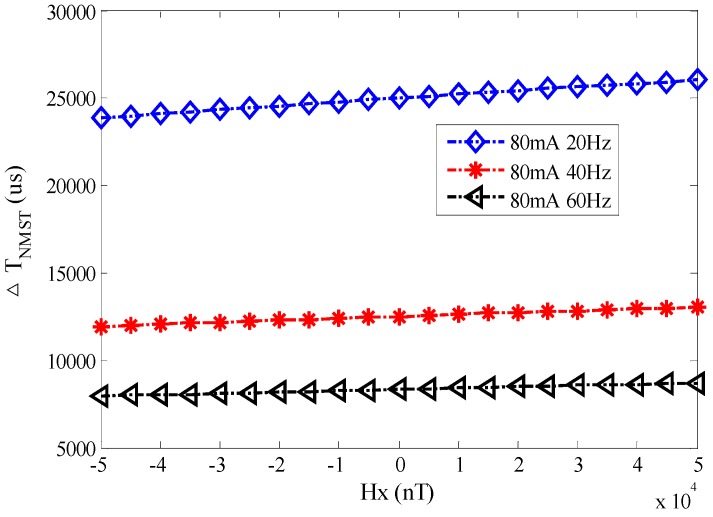
The curve between *H_x_* and Δ*T_NMST_* with different excitation frequencies.

(b) When the excitation current is 80 mA, the excitation frequency changes from 20 Hz to 60 Hz with a 20 Hz interval. *H*_x_ is the same as mentioned above. Figure 12 shows the output time difference Δ*T_NMST_* of the RTD-fluxgate which is actually measured with different excitation frequencies.

According to the least square fitting method, the fitting curves between Δ*T_NMST_* and *H_x_* with different excitation frequencies, *f*_1_ = 20 Hz, *f*_2_ = 40 Hz, and *f*_3_ = 60 Hz, are as follows:

Δ*T_NMST_*_1_ = 0.0218 × *H_x_* + 2.50 × 10^4^ (23)

Δ*T_NMST_*_2_ = 0.0109 × *H_x_* + 1.25 × 10^4^ (24)

Δ*T_NMST_*_3_ = 0.0074 × *H_x_* + 8.34 × 10^3^ (25)

From the Equations above, the sensitivities of different excitation frequencies are *S_NMST_*_1_ = 0.0218 μs/nT, *S_NMST_*_2_ = 0.0109 μs/nT, and *S_NMST_*_3_ = 0.0074 μs/nT. When the excitation frequency *f* is smaller, the sensitivity *S_NMST_* is greater. In Figure 10 and Figure 12, the experimental results validate that the sensitivity *S_NMST_* is inversely proportional to the amplitude *H_m_* and frequency *f* of the excitation magnetic field.

(2) Stability Analysis

**Figure 13:**
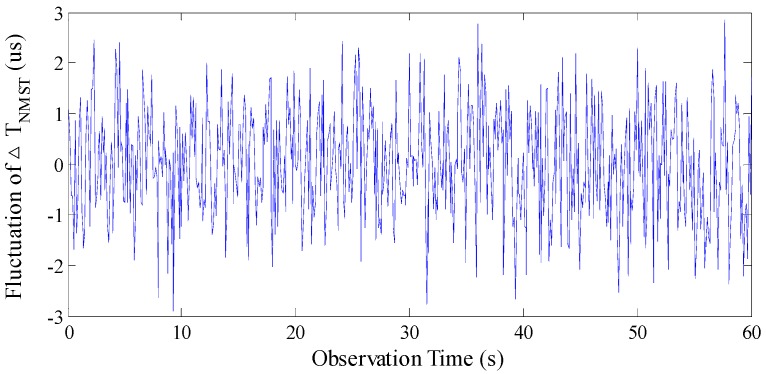
The fluctuation of Δ*T_NMST_* by using the NMST readout scheme.

**Figure 14:**
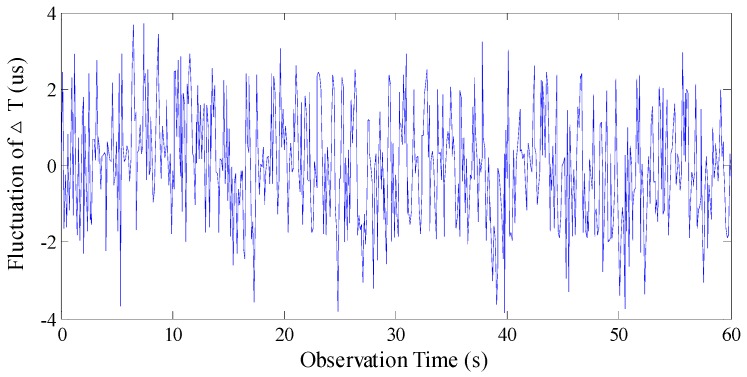
The fluctuation of Δ*T* by using the BMSTD readout scheme.

Analysis was performed under the conditions of an excitation magnetic field with parameters *I* = 80 mA, *f* = 60 Hz, and *H_x_* = 25,000 nT. To compare the stability of the two readout methods effectively, the time of observation is 60 s. The fluctuations in time difference by using the NMST and BMSTD readout methods are shown in Figure 13 and Figure 14, respectively. Because the observation time is longer, the data of time difference fluctuations are larger. We only present the data of 3 s among 60 s in Table 1.

As is illustrated in Figure 13 and Figure 14, by using the NMST readout scheme, the standard deviation of Δ*T_NMST_* is 1.086 μs and the fluctuation of Δ*T_NMST_* is 5.780 μs. By using the BMSTD readout scheme, the standard deviation of Δ*T* is 1.465 μs and the fluctuation of Δ*T* is 7.559 μs. The comparison between the two readout methods indicates that the standard deviation and the fluctuation of the time difference are reduced by 36% and 32%, respectively, by adopting the NMST readout scheme.

In order to process the time difference dynamically in real time, in this paper, the variable coefficient Pauta criterion and equal-weight endpoint smoothing are combined to form a hybrid time difference processing algorithm. The specific procedure is as follows:

a Every *n* Δ*T_NMST_* forms an array *N*_i_, and then the mean $\bar{N_{i}}$ and variance *σ*_i_ of each array need calculating in turn.
b The amount of effective data after processing is more than 3/4 times of the amount of data before processing. When |Δ*T*_NMST(i−1)n+j_ − $\bar{N_{i}}$| > *kσ*_i_, Δ*T*_NMST(i−1)n+j_ is considered as gross error, therefore, the value is replaced by the mean of the array; when |Δ*T*_NMST(i−1)n+j_ − $\bar{N_{i}}$| < *kσ*_i_, this value is reserved. A new time difference array *N*’ = *N*’_1_ + *N*’_2_ + … *N*’*_i_* is formed.
c At the initial point of the new data sequence *N*’, 50 consecutive data points are processed with equal weight endpoints at a time through the equation $\Delta T_{NMSTk}=\frac{1}{l}\sum_{i=0}^{l-1}\Delta T_{NMST(k+i)}$, *k* = 1, 2, 3, …, *n* × *I − l* + 1. In this paper, the sequence *N*’ is processed twice.

**Figure 15:**
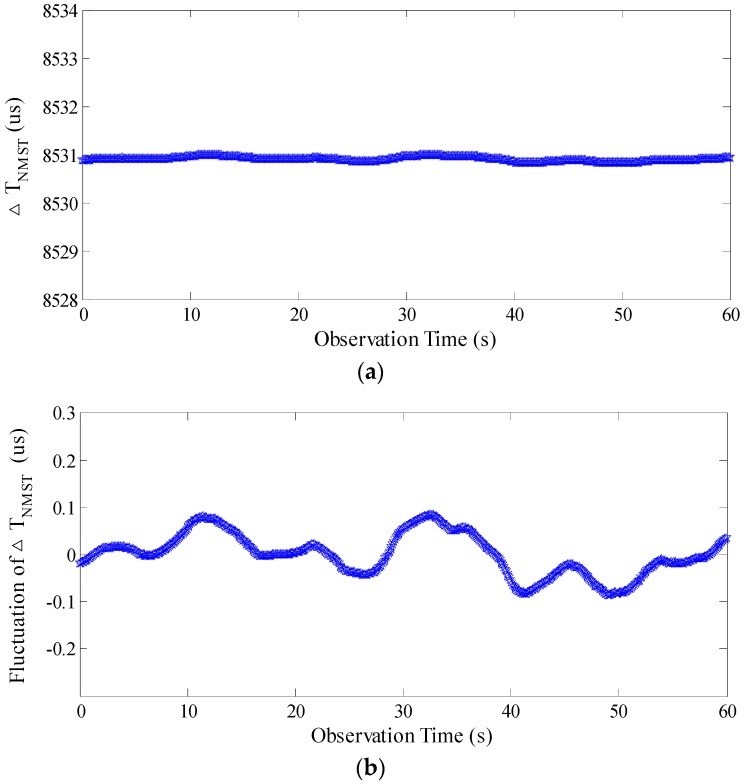
The Δ*T_NMST_* and fluctuation after processing by the hybrid algorithm. (**a**) Δ*T_NMST_* after hybrid algorithm processing; (**b**) The fluctuation of Δ*T_NMST_* after hybrid algorithm processing.

After hybrid algorithm processing, the standard deviation of Δ*T_NMST_* is reduced to 0.044 μs and the fluctuation is reduced to 0.170 μs, as is shown in Figure 15.

## 5. Conclusions

On the basis of the working principle of RTD-fluxgate sensors, the influence of the induction signal noise on the time difference reading is analyzed. A readout method is proposed in which the excitation and induction signals are combined to read the negative magnetic saturation time. A mathematical model of the RTD-fluxgate sensor output response between Δ*T_NMST_* and *H_x_* is established. The NMST readout scheme, which is proposed in this paper, is compared with the BMSTD readout scheme. The experimental results validate the effectiveness of the readout method. The standard deviation and the fluctuation of the time difference are reduced by 36% and 32%, respectively. This technique of RTD-fluxgate sensor usage can reduce the noise influence and improve the stability of time difference measurement. After Δ*T_NMST_* is processed by the time difference hybrid algorithm, the fluctuation can be stabilized within ±0.1 μs, so the accuracy of RTD-fluxgate measurement is improved further. The NMST readout method is suitable for RTD-fluxgate detection of weak magnetic fields.

## Figures and Tables

**Table 1:** Time difference fluctuations of 3 s by using two readout methods.

Time (s)	Fluctuations of Δ*T_NMST_* (μs)	Fluctuations of Δ*T* (μs)	Time (s)	Fluctuations of Δ*T_NMST_* (μs)	Fluctuations of Δ*T* (μs)
36.1	0.884	0.719	37.6	0.882	−0.158
36.2	−0.755	−1.221	37.7	1.215	0.219
36.3	0.913	0.180	37.8	0.283	3.219
36.4	2.364	−1.642	37.9	0.784	0.678
36.5	0.136	−2.061	38.0	1.251	0.459
36.6	1.765	−1.275	38.1	0.403	−1.949
36.7	1.443	−0.239	38.2	0.672	−0.648
36.8	−0.992	−0.334	38.3	−2.198	0.197
36.9	0.748	1.552	38.4	−1.016	−1.426
37.0	−0.095	−0.367	38.5	1.474	−0.968
37.1	0.334	2.292	38.6	−0.228	−2.057
37.2	−0.455	0.788	38.7	1.563	−3.038
37.3	−1.107	0.199	38.8	0.486	−1.587
37.4	0.895	−1.476	38.9	1.040	−1.955
37.5	0.483	0.177	39.0	0.335	−3.651
